# Carbonate-silicate cycle predictions of Earth-like planetary climates and testing the habitable zone concept

**DOI:** 10.1038/s41467-020-19896-2

**Published:** 2020-12-01

**Authors:** Owen R. Lehmer, David C. Catling, Joshua Krissansen-Totton

**Affiliations:** 1 MS 239-4, Space Science Division, NASA Ames Research Center, Moffett Field, CA 94035 USA; 2 Department of Earth and Space Sciences/Astrobiology Program, University of Washington, Box 351310, Seattle, WA 98195 USA; 3 Virtual Planetary Laboratory at the University of Washington, Seattle, WA 98195 USA; 4 Department of Astronomy and Astrophysics, MS UCO/Lick Observatory, 1156 High Street, Santa Cruz, CA 95064 USA

**Keywords:** Astrobiology, Atmospheric chemistry, Exoplanets

## Abstract

In the conventional habitable zone (HZ) concept, a CO_2_-H_2_O greenhouse maintains surface liquid water. Through the water-mediated carbonate-silicate weathering cycle, atmospheric CO_2_ partial pressure (pCO_2_) responds to changes in surface temperature, stabilizing the climate over geologic timescales. We show that this weathering feedback ought to produce a log-linear relationship between pCO_2_ and incident flux on Earth-like planets in the HZ. However, this trend has scatter because geophysical and physicochemical parameters can vary, such as land area for weathering and CO_2_ outgassing fluxes. Using a coupled climate and carbonate-silicate weathering model, we quantify the likely scatter in pCO_2_ with orbital distance throughout the HZ. From this dispersion, we predict a two-dimensional relationship between incident flux and pCO_2_ in the HZ and show that it could be detected from at least 83 (2*σ*) Earth-like exoplanet observations. If fewer Earth-like exoplanets are observed, testing the HZ hypothesis from this relationship could be difficult.

## Abstract (web summary)

In the habitable zone concept, a planet’s carbon dioxide-water greenhouse maintains surface liquid water. Here, the authors estimate how many Earthlike exoplanets are needed to detect a relationship between stellar flux and the atmospheric carbon dioxide predicted by carbon cycle modeling.

## Introduction

Newton first alluded to the concept of a stellar habitable zone (HZ) in his 1687 Principia^1^ by noting that Earth’s liquid water would vaporize or freeze at the orbits of Mercury and Saturn, respectively^2^. Later, Whewell noted that “the Earth’s orbit is in the temperate zone of the Solar System”^3^. Since then, the definition of the stellar HZ has been refined, reaching its modern incarnation based on climate models^4,5^.

Current HZ calculations^6^ find that around a Sun-like star, an Earth-like planet could remain habitable between 0.97 and 1.70 AU. The inner edge of the HZ is set by loss of surface water and the outer edge is set by the maximum greenhouse of a CO_2_ atmosphere where extensive CO_2_ condensation and increased Rayleigh scattering prevent any further greenhouse warming from CO_2_ (refs. ^6,7^). This definition of the HZ only considers H_2_O and CO_2_ as greenhouse gases, so Earth-like planets warmed by other greenhouse gases (e.g., H_2_ or CH_4_) could remain habitable at bigger orbital distances^5,8,9^. However, CH_4_-rich atmospheres in the HZ may not be possible without life to generate substantial CH_4_ (refs. ^10,11^). In addition, more complex climate models have shown the HZ might extend to smaller orbital distances, perhaps interior to Venus’ orbit, with appropriate planetary conditions^12–15^.

Residing within the HZ does not guarantee habitable surface conditions. Crucially, greenhouse gas abundances (and planetary albedo) must conspire to produce clement surface conditions. For example, by most estimates, Mars resides within the Sun’s HZ but is not habitable because there is insufficient greenhouse warming from CO_2_, in part because of the lack of volcanic outgassing of CO_2_. Thus, considering the planetary processes that control atmospheric CO_2_ abundances on Earth-like planets in the HZ is necessary to constrain planetary habitability.

The prevailing hypothesis is that CO_2_ levels are controlled by a weathering thermostat^16^. This can explain why Earth has maintained a clement surface throughout its history despite the  ~30% brightening of the Sun over the past  ~4.5 Gyr^17–21^. The changing luminosity of the Sun with time is similar to moving a planet through the HZ, and so the same CO_2_ weathering process responsible for maintaining habitability on the Earth through time, the carbonate–silicate weathering cycle, may similarly stabilize planetary climates within the HZ.

In the carbonate–silicate cycle, atmospheric CO_2_ dissolves in water and weathers silicates on both the continents and seafloor, which releases cations and anions^16,22–27^. On the continents, the weathering products, including dissolved SiO_2_, HCO${\,}_{3}^{-}$, and Ca^++^, wash into the oceans where the HCO${\,}_{3}^{-}$ combine with cations like Ca^++^ to create CaCO_3_, which precipitates out of solution. The net process converts atmospheric CO_2_ into marine carbonate minerals (i.e., CaCO_3_). Also, seafloor weathering occurs when seawater releases Ca^++^ ions from the seafloor basalt and CaCO_3_ precipitates in pores and veins. Subsequently, the carbonates within sediments and altered seafloor can be subducted.

Carbon returns to the atmosphere from outgassing. If CO_2_ outgassing increases above the steady-state outgassing rate, a planet’s surface temperature rises. This leads to increased rainfall and continental weathering as well as potentially warmer deep-sea temperatures and more seafloor weathering^21,24,28^. Increased weathering draws down atmospheric CO_2_ and stabilizes the climate over   ~10^6^-year timescales on habitable, Earth-like planets^29^.

One- and three-dimensional (1D, 3D) climate calculations of HZ limits^4,6,14^ assume that a carbonate–silicate weathering cycle is functioning but do not explicitly include it. The assumed presence of the carbonate–silicate cycle would predict that atmospheric CO_2_ of Earth-like planets increases with orbital distance in the HZ^4,6,29^. In particular, future telescopic observations, e.g., NASA’s Habitable Exoplanet Imaging Mission (HabEx)^30^ and Large Ultraviolet Optical Infrared Surveyor (LUVOIR)^31^, could search for the CO_2_ trend to test the HZ hypothesis^32–34^. Previous studies^29,35^ have suggested the carbonate–silicate weathering cycle could alter predictions of pCO_2_ in the HZ, but it is important to know the exact relationship we are looking for. Also, while an increase of pCO_2_ with orbital distance in the HZ may be true if all Earth-like exoplanets have the exact same properties as the modern Earth, the trend becomes less certain if HZ planetary characteristics deviate from those of the modern Earth. There could be considerable variability in atmospheric CO_2_ throughout the HZ, perhaps even enough to obscure a monotonic trend with orbital distance.

Here, we show that uncertain physicochemical and geophysical parameters in the carbonate–silicate weathering cycle^26^ cause scatter in pCO_2_ with orbital distance. We then demonstrate that future telescopes must observe at least 83 (2*σ*) HZ planetary atmospheres to confidently detect our predicted relationship between atmospheric CO_2_ and orbital distance, and confirm the HZ hypothesis.

## Results

### Stable pCO_2_ abundances from our numerical model

We use a coupled climate and carbonate–silicate weathering model (see Methods, subsection “Numerical carbonate–silicate cycle modeling”) to explore pCO_2_ on Earth-like planets in the HZ. The model considers numerous planetary properties, listed in Table 1, and their effect on the carbonate–silicate weathering cycle to calculate a planet’s steady-state pCO_2_ and surface temperature. If the globally averaged, steady-state surface temperature is below 248 K, we assume the planet is completely frozen and uninhabitable at the surface, as shown by 3D climate models^36^. Similarly, we assume planets are uninhabitable beyond 355 K, above which surface water would be rapidly lost to space^37^ (see Methods, subsection “Numerical carbonate–silicate cycle modeling” for additional details on these assumed temperature constraints).

**Table 1:** Parameter ranges for our numerical model.

Parameter	Parameter description	Range	Scaling	Units
${F}_{{\rm{out}}}^{{\rm{mod}}}$	Modern CO_2_ outgassing flux	6–10		Tmol C yr^−1^
*n*	Carbonate precipitation coefficient	1–2.5	$\propto {\left[{{\rm{CO}}}_{3}^{2-}\right]}^{n}$	
*x*	Modern seafloor dissolution relative to precipitation	0.5–1.5	$\propto x{F}_{{\rm{out}}}^{{\rm{mod}}}$	
*T*_e_	E-folding temperature factor for continental weathering	10–40		K
*α*	Power law exponent for CO_2_ dependence of continental silicate weathering	0.1–0.5	$\propto {\left({{\rm{pCO}}}_{2}\right)}^{\alpha }$	
*ξ*	Power law exponent for CO_2_ dependence of continental carbonate weathering	0.1–0.5	$\propto {\left({{\rm{pCO}}}_{2}\right)}^{\xi }$	
*f*_land_	Land fraction compared to modern Earth	0–1		
*S*_thick_	Ocean sediment thickness relative to modern Earth	0.2–1		
${F}_{{\rm{carb}}}^{{\rm{mod}}}$	Modern continental carbonate weathering	7–14		Tmol C yr^−1^
*f*_bio_	Biological weathering compared to modern Earth	0–1		
${a}_{{\rm{grad}}}$	Surface to deep ocean temperature gradient scaling	0.8–1.4	$\propto {a}_{{\rm{grad}}}{T}_{{\rm{s}}}$	
*γ*	Power law exponent for pH dependence of seafloor dissolution	0–0.5	$\propto {\left(\left[{{\rm{H}}}^{+}\right]\right)}^{\gamma }$	
*β*	Power law exponent for seafloor spreading rate	0–0.2	∝ *Q*^*β*^	
*m*	Exponent for outgassing dependence on crustal production	1–2	∝ *Q*^*m*^	
*E*_bas_	Seafloor dissolution activation energy	60–100		kJ mol^−1^
*n*_out_	Exponent for internal heat with time	0–0.73	see Eq. (10)	
*τ*	Planet age (see Eq. (10))*	0–10		Gyr
*S*	Incident flux relative to modern Earth*	0.35–1.05		

Parameters are dimensionless unless otherwise described. The fourth column shows how scaling parameters impact the model, where *T*_s_ is the surface temperature in K and *Q* is the internal heat of the planet relative to the modern Earth (see Eq. (10) for *Q*). Unless otherwise noted, each parameter range is justified in the original model derivation for the Earth through time^21^.

*The justification for this parameter is given in the Methods, subsection “Numerical carbonate–silicate cycle modeling”.

**Fig. 1:**
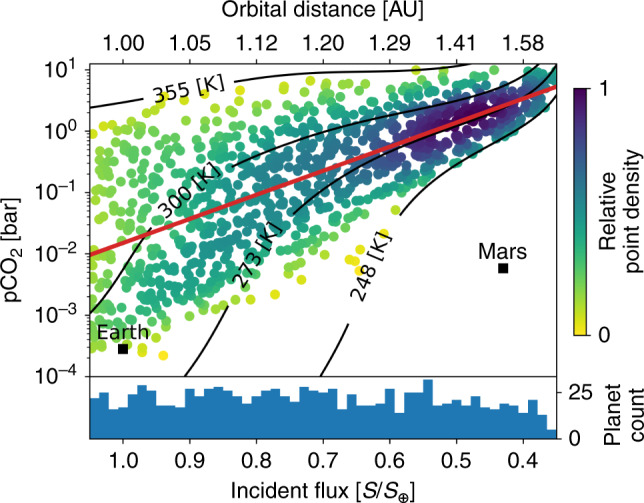
The expected distribution of stable, Earth-like exoplanet climates from our habitable zone weathering model. The horizontal axis shows incident flux, *S*, normalized to the solar constant (*S*_⊕_) and the corresponding orbital distance in Astronomical Units (AU) above the plot. The vertical axis shows the atmospheric CO_2_ partial pressure (pCO_2_) in bar. Each point represents a climate in steady state. The black labeled contours show the mean global surface temperature for the given pCO_2_ and incident flux. The white region below the 248 K contour is where our model assumption of a liquid ocean is no longer plausible so no planets are shown in that region. Above the 355 K contour, Earth-like planets are too hot to retain their liquid oceans for billions of years. Similar to the frozen planets, such hot planets are not considered habitable. Modern Earth and Mars are shown by black squares. The blue histogram at the bottom of the figure shows the number of stable planets in each incident flux bin. The color of each simulated planet shows the relative point density in the plot at that location. The color was calculated using a kernel-density estimate with Gaussian kernels and rescaled from 0 to 1. A color value of 0 represents the lowest relative point density, 1 the highest. The log-linear line of best fit between pCO_2_ and *S* is shown in red. The slope of the red, best fit line is 3.92 ± 0.24 (95%) with units −${\mathrm{log}\,}_{10}$(pCO_2_ [bar])/(*S*/*S*_⊕_). Our model predicts that atmospheric CO_2_ should increase with orbital distance in the HZ.

We randomly generated 1050 habitable, stable, Earth-like exoplanet climates using uniform distributions of the model parameters in Table 1. A total of 1200 random, initial parameter combinations were considered but we eliminated those that resulted in planets that froze completely or were too hot to retain their surface oceans. As colored dots, Fig. 1 shows habitable, steady-state solutions.

Our model predicts that atmospheric CO_2_ abundances should broadly increase and narrow in their spread with orbital distance in the HZ (Fig. 1), consistent with other models of CO_2_ in the HZ^29,38^. As justified next in section “Habitable zone climate theory revisited”, the scatter is about a nominal linear trend between incident flux, *S*, and log(pCO_2_), which is different from a non-linear trend in models that assume a constant surface temperature in the HZ from negative feedbacks^32,34^ but do not actually model the carbonate–silicate feedbacks. If future missions are to test the HZ concept by searching for a trend between incident flux, *S*, and pCO_2_ (refs. ^32–34^), they could search for the fundamental *S*–pCO_2_ relationship shown in Fig. 1.

Below, we show that a log-linear relationship between pCO_2_ and *S* may be the default in the HZ if Earth-like carbonate–silicate weathering is ubiquitous on habitable planets. In fact, the trend is elucidated by combining climate theory with carbonate–silicate cycle theory in what follows.

### Habitable zone climate theory revisited

**Fig. 2:**
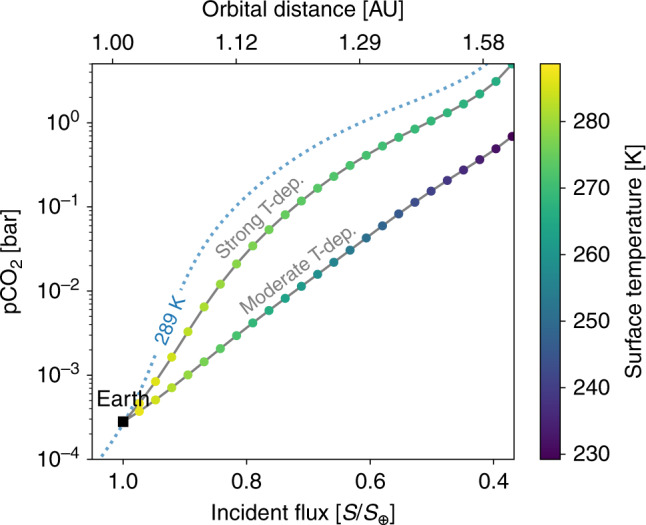
The relationship between incident flux and atmospheric CO_2_ for Earth-like planets regulated by a carbonate–silicate weathering cycle. The horizontal axis shows incident flux, *S*, normalized to the solar constant (*S*_⊕_) and the corresponding orbital distance in Astronomical Units (AU) above the plot. The vertical axis shows the atmospheric CO_2_ partial pressure (pCO_2_) in bar. The dotted blue curve labeled 289 K shows the pCO_2_ value required to maintain a 289 K surface temperature for the given incident flux, *S*. The conventional assumption of CO_2_ in the HZ stipulates that pCO_2_ will adjust to maintain a temperate or even constant surface temperature. Under this assumption, moving the modern Earth (labeled black square) outward in the HZ would have the planet approximately follow the dotted blue 289 K contour. The colored points and gray curves show the modern Earth moving outward in the HZ with a functioning carbonate–silicate weathering cycle, calculated from Eq. (6). We consider two temperature and pCO_2_ dependencies for continental weathering in this plot. The strong temperature dependence contour (labeled Strong T-dep.), uses a temperature and pCO_2_-dependent weathering factor of *αT*_e_ = 2.3, which implies a strong temperature feedback on continental weathering compared to the pCO_2_ feedback (see Eq. (7)). The moderate temperature dependence contour (labeled Moderate T-dep.), uses a temperature and pCO_2_-dependent weathering factor of *αT*_e_ = 7.5. These two values for *αT*_e_ result in two different paths the Earth can take as it moves outward in the HZ. The planet color shows the mean surface temperature. Log-linear fits to the colored points of the Strong T-dep. and Moderate T-dep. contours have *r*^2^ values of 0.959 and 0.999, respectively. Thus, even for a strong temperature dependence of continental weathering, our coupled climate and weathering model predicts an approximately log-linear relationship between incident flux and pCO_2_ on Earth-like planets in the HZ.

A conventional assumption is that the carbonate–silicate weathering cycle will approximately maintain a stable, temperate surface temperature for an Earth-like planet moved about in the HZ^6,7,39^ or even a constant temperature^32,34^. Thus, if we moved the modern Earth outward in the HZ, the smaller incident flux would initially cause the planet to cool. The cooler temperature would lower the CO_2_ weathering rate causing CO_2_ to accumulate in the atmosphere until the temperature returned to its nominal value of 289 K. Figure 2 shows this scenario with the dotted blue 289 K contour, which gives the pCO_2_ value required to maintain a 289-K surface temperature for the modern Earth as it moves about the HZ. The line was calculated from a radiative-convective climate model described in the Methods below, subsection “Habitable zone 1D climate model” (see Eq. (8)).

The constant, 289 K surface temperature contour in Fig. 2 is a non-linear relationship between incident flux, *S*, and log(pCO_2_) but it does not consider the temperature and pCO_2_ feedbacks inherent to the carbonate–silicate weathering cycle. We demonstrate that if these feedbacks are taken into account, surface temperature declines with orbital distance, as mentioned in previous work^29^, and the relationship between *S* and log(pCO_2_) is actually approximately linear for Earth-like planets in the HZ.

If Bond albedo is fixed, the surface temperature, *T*_s_, for an Earth-like planet in steady state varies approximately linearly with incident flux, *S*^5,40,41^. This linear relationship between *T*_s_ and *S* arises from energy balance and from water vapor feedback and can be expressed as

1 $${F}_{{\rm{SOL}}}={F}_{{\rm{OLR}}}=\left(\frac{1-{A}_{{\rm{B}}}}{4}\right)S=a+b{T}_{{\rm{s}}},$$

where *F*_SOL_ is the incoming solar radiation flux, *F*_OLR_ the outgoing long-wavelength radiation flux, *A*_B_ the Bond albedo, and *a* and *b* are empirical constants. From satellite measurements of the modern Earth and radiative calculations, for *T*_s_ in K, the empirical constants in Eq. (1) are approximately *a* = −370 W m^−2^ and *b* = 2.2 W m^−2^ K^−1^ (ref. ^41^).

Solving for *T*_s_ in Eq. (1), the surface temperature is given by

2 $${T}_{{\rm{s}}}=\left(\frac{1-{A}_{{\rm{B}}}}{4b}\right)S-\frac{a}{b}.$$

Under the conventional assumption that the HZ is regulated by a CO_2_–H_2_O greenhouse effect where H_2_O concentrations respond to changes in pCO_2_, the temperature offset in Eq. (2),  −*a*/*b*, is a function of pCO_2_. Thus, surface temperature, as a function of *S* and pCO_2_, is given by

3 $${T}_{{\rm{s}}}\left(S,{{\rm{pCO}}}_{2}\right)=\left(\frac{1-{A}_{{\rm{B}}}}{4b}\right)S+f\left({{\rm{pCO}}}_{2}\right),$$

where $f\left({{\rm{pCO}}}_{2}\right)$ is a function that depends on pCO_2_. For the modern Earth at 1 AU, *f*(pCO_2_) = −*a*/*b*. For pCO_2_ ≤ 0.1 bar, the CO_2_ greenhouse effect is logarithmic in pCO_2_, i.e., $f({{\rm{pCO}}}_{2})\propto \mathrm{ln}\,({{\rm{pCO}}}_{2})$^42,43^. Above   ~0.1 bar, weaker CO_2_ absorption features become important and *f*(pCO_2_) deviates from $\propto \mathrm{ln}\,({{\rm{pCO}}}_{2})$^43,44^.

As pCO_2_ increases for an Earth-like planet moved outward in the HZ, the surface temperature will follow Eq. (3) while the rate at which CO_2_ is removed from the atmosphere will adjust according to the carbonate–silicate weathering feedback. Quantitatively, the pCO_2_- and *T*_s_-dependent flux of CO_2_ removal due to the continental weathering flux, *F*_w_ (in mol CO_2_ per unit time) is described by

4 $${F}_{{\rm{w}}}=\rho {\left(\frac{{{\rm{pCO}}}_{2}}{{{\rm{pCO}}}_{2}^{{\rm{mod}}}}\right)}^{\alpha }\exp \left(\frac{{T}_{{\rm{s}}}\left(S,{{\rm{pCO}}}_{2}\right)-{T}_{{\rm{s}}}^{{\rm{mod}}}}{{T}_{{\rm{e}}}}\right),$$

where *ρ* is a constant determined by the continental weathering properties of the modern Earth, *α* a dimensionless constant between 0.1 and 0.5 and regulates the pCO_2_ dependence of continental silicate weathering, *T*_e_ a constant between 10 K and 40 K and represents the e-folding temperature dependence of continental weathering. The range for *T*_e_ has been empirically constrained for the surface temperatures relevant to habitable, Earth-like planets from lab measurements and Phanerozoic geologic constraints^16,26^. Finally, pCO${\,}_{2}^{{\rm{mod}}}=288\times 1{0}^{-6}$ bar and ${T}_{{\rm{s}}}^{{\rm{mod}}}=289$ K are the modern Earth’s preindustrial pCO_2_ and surface temperature, respectively^21^.

The range for *α* on the Earth was determined empirically from geologic constraints over the past 100 Myr^26^. We assume that this derived range for *α* applies to the Earth through time^21,45,46^ and the Earth-like exoplanets modeled here that have a carbonate–silicate cycle. However, better proxy data for the ancient Earth or observing the carbonate–silicate cycle on habitable exoplanets^32,34^ may be necessary to understand if the assumed range for *α* applies more generally to habitable planets.

In Eq. (4), we assume seafloor weathering is negligible, which is a reasonable approximation for the modern Earth^21^, and illustrative for our purposes of deriving a simple, analytic relationship between *S* and pCO_2_. Here, we seek to predict the behavior of the modern Earth in the HZ to gain intuitive understanding, whereas in our numerical model we consider a broad range of properties for Earth-like planets on which seafloor weathering may be important.

The modern Earth, and all Earth-like planets considered in this work, are assumed to be in steady state, in which the flux of CO_2_ from volcanic outgassing is equal to the rate of CO_2_ removal from weathering, *F*_w_. If we assume a HZ planet with CO_2_ outgassing the same as the modern Earth’s, *F*_w_ remains constant despite changes in *S* and pCO_2_. Setting ${T}_{{\rm{s}}}\left(S,{{\rm{pCO}}}_{2}\right)={T}_{{\rm{s}}}^{{\rm{mod}}}$ and ${{\rm{pCO}}}_{2}={{\rm{pCO}}}_{2}^{{\rm{mod}}}$ for the modern Earth, from Eq. (4), we see that *F*_w_ = *ρ* and

5 $$1={\left(\frac{{{\rm{pCO}}}_{2}}{{{\rm{pCO}}}_{2}^{{\rm{mod}}}}\right)}^{\alpha }\exp \left(\frac{{T}_{{\rm{s}}}\left(S,{{\rm{pCO}}}_{2}\right)-{T}_{{\rm{s}}}^{{\rm{mod}}}}{{T}_{{\rm{e}}}}\right).$$

Equation (5) must hold for a modern Earth within the HZ. If it did not, *F*_w_ would not balance CO_2_ outgassing, which would result in either complete CO_2_ removal, or CO_2_ accumulation without bound.

Solving for ${T}_{{\rm{s}}}\left(S,{{\rm{pCO}}}_{2}\right)$ in Eq. (5), we find

6 $${T}_{{\rm{s}}}\left(S,{{\rm{pCO}}}_{2}\right)={T}_{{\rm{s}}}^{{\rm{mod}}}-\alpha {T}_{{\rm{e}}}\mathrm{ln}\,\left(\frac{{{\rm{pCO}}}_{2}}{{{\rm{pCO}}}_{2}^{{\rm{mod}}}}\right).$$

Equating Eq. (6) to Eq. (3) and rearranging gives

7 $$\left(\frac{1-{A}_{{\rm{B}}}}{4b}\right)S={T}_{{\rm{s}}}^{{\rm{mod}}}-\left[\alpha {T}_{{\rm{e}}}{\mathrm{ln}}\,\left(\frac{{{\rm{pCO}}}_{2}}{{{\rm{pCO}}}_{2}^{{\rm{mod}}}}\right)+f\left({{\rm{pCO}}}_{2}\right)\right].$$

If $f({{\rm{pCO}}}_{2})\propto \mathrm{ln}\,({{\rm{pCO}}}_{2})$, which is the case for pCO_2_ ≤ 0.1 bar^43,44^, then $S\propto -\mathrm{ln}\,({{\rm{pCO}}}_{2})$. However, even if *f*(pCO_2_) deviates from log-linearity with pCO_2_, *S* will become increasingly log-linear with pCO_2_ as *αT*_e_ increases. In Eq. (7), increasing *αT*_e_ will cause the $\mathrm{ln}\,({{\rm{pCO}}}_{2})$ term to dominate the relationship between *S* and pCO_2_. Intuitively, increasing *αT*_e_ decreases the temperature dependence of continental weathering relative to its pCO_2_ dependence. Note that bigger *T*_e_ reduces the temperature dependence of continental weathering while bigger *α* increases the pCO_2_ dependence of continental weathering (Eq. (4)).

In addition to predicting a linear relationship between ${\mathrm{log}}\left({\mathrm{pCO}}_{2}\right)$ and *S*, the carbonate–silicate cycle implies that moving an Earth-like planet outward in the HZ will cause ${T}_{{\rm{s}}}\left(S,{{\rm{pCO}}}_{2}\right)$ to decrease. For increasing orbital distance, pCO_2_ must increase for ${T}_{{\rm{s}}}\left(S,{{\rm{pCO}}}_{2}\right)$ to increase in the HZ. From Eq. (5), pCO_2_ will be greater than pCO${\,}_{2}^{{\rm{mod}}}$ in such cases so ${T}_{{\rm{s}}}\left(S,{{\rm{pCO}}}_{2}\right)$ must be less than ${T}_{{\rm{s}}}^{{\rm{mod}}}$. This decrease in ${T}_{{\rm{s}}}\left(S,{{\rm{pCO}}}_{2}\right)$ as *S* decreases is shown in Fig. 2. Physically, the power law dependence of weathering on pCO_2_ can balance volcanic outgassing at lower surface temperatures in the outer HZ.

 Figure 2 shows the approximately log-linear relationship between pCO_2_ and *S* for the modern Earth moved outward in the HZ. The gray lines and colored circles in Fig. 2 show the expected pCO_2_ value for the given incident flux *S*, calculated from Eq. (6). For each *S* value in Fig. 2, Eq. (6) was solved for pCO_2_ by using Eq. (8), the polynomial fit for surface temperature based on a 1D climate model (described in the Methods, subsection “Habitable zone 1D climate model”), assuming values of *αT*_e_.

The value of *αT*_e_ affects the slope of the relationship between *S* and pCO_2_ due to the carbonate–silicate weathering cycle, shown in Fig. 2. From above, the ranges for *α* and *T*_e_ are 0.1 ≤ *α* ≤ 0.5 and 10 ≤ *T*_e_ ≤ 40 (ref. ^21^), so 1 ≤ *αT*_e_ ≤ 20. If we consider uniform distributions of *α* and *T*_e_, then roughly 95% of *αT*_e_ values will be greater than 2.3. If *α* = 0.23 and *T*_e_ = 10 K then *αT*_e_ =  2.3, which is used for the Strong T-dep. curve in Fig. 2. The mean of each parameter, *α* = 0.3 and *T*_e_ = 25 K gives *αT*_e_ = 7.5, which corresponds to the Moderate T-dep. curve in Fig. 2. For *αT*_e_ ≤ 2.3 the colored points and gray curves become increasingly similar to the constant 289 K surface temperature contour in Fig. 2. However, for uniform distributions of *α* and *T*_e_,   ~95% of *αT*_e_ values are greater than 2.3, so an approximately log-linear relationship between *S* and $\mathrm{log}\,({{\rm{pCO}}}_{2})$ is the default expectation for Earth-like planets in the HZ.

### Observational uncertainty for pCO_2_ in the HZ

In the log-linear fit shown as the solid red line in Fig. 1, which is the expected relationship between pCO_2_ and *S* that we have derived above, the *r*^2^-value is 0.49. Thus, about half the variance in log(pCO_2_) is described by changes in incident flux. The slope is 3.92 ± 0.24 (95%) with units −${\mathrm{log}\,}_{10}$(pCO_2_ [bar])/(*S*/*S*_⊕_), so our model predicts a trend of increasing atmospheric CO_2_ with orbital distance, which future missions might detect^32–34^. However, there is sufficient spread in our simulated planets that confirming the HZ hypothesis from such a trend may be difficult.

This difficulty is readily seen if we consider a log-uniform distribution for pCO_2_ on Earth-like planets in the HZ. If we randomly generate 1050 such planets, where 10^−4^ ≤ pCO_2_ ≤ 10 bar is sampled log-uniformly, 0.35 ≤ *S* ≤ 1.05 is sampled uniformly, and impose the same constraints on surface temperature for habitability as in Fig. 1, then the log-linear line of best fit through the uniform planet data has a slope of 3.76 ± 0.465 (95%) with units −${\mathrm{log}\,}_{10}$(pCO_2_ [bar])/(*S*/*S*_⊕_). Thus, measuring just the log-linear trend between pCO_2_ and *S* in the HZ is unlikely to test the HZ hypothesis as this measurement cannot confidently detect the presence of the carbonate–silicate weathering cycle—it is indistinguishable from that of randomly distributed pCO_2_ between the surface temperature limits for habitability.

The inability to differentiate between the log-linear trends for weathering-mediated and random pCO_2_ vs *S* in the HZ is due to the assumed surface temperature constraints we impose in our model (between 248 and 355 K for planets in the HZ, see Methods, subsection “Numerical carbonate–silicate cycle modeling”). Such temperature constraints are necessary as the carbonate–silicate weathering cycle can only operate when water, as liquid, is present at the planetary surface. Even without the carbonate–silicate weathering cycle, a minimum surface temperature for habitable planets, which must exist, will result in increasing pCO_2_ with orbital distance, as shown by the constant temperature contours in Fig. 1.

To test the HZ hypothesis, we propose searching for the two-dimensional (2D) distribution of planets in the *S*-pCO_2_ phase space that arises from the carbonate–silicate weathering cycle. This *S*-log(pCO_2_) relationship is shown by the point density in Fig. 1, where the distribution of habitable, stable planets is not log-uniformly distributed over pCO_2_. Rather, around the best-fit line, there is an abundance of planets in the outer HZ at high pCO_2_, a dearth of low pCO_2_ planets between   ~0.9 and   ~0.7 *S*/*S*_⊕_, and few high-pCO_2_ planets throughout the HZ compared to the log-uniform pCO_2_ case. These differences are expected features of the carbonate–silicate weathering cycle due to the temperature- and pCO_2_-dependent nature of the weathering feedback. Recall from section “Habitable zone climate theory revisited”  that, as *S* decreases, the lowered temperature will reduce weathering causing pCO_2_ to increase. This results in the lack of low-pCO_2_ planets in the middle of the HZ and the high abundance of habitable planets in the outer HZ (purple shaded region in Fig. 1). Similarly, for large pCO_2_, the temperature is warmer and pCO_2_ higher than that of modern Earth so the carbonate–silicate weathering cycle acts to lower pCO_2_, which reduces the number of high-pCO_2_ planets throughout the HZ relative to the outer HZ.

To detect the prevalence of the carbonate–silicate weathering cycle and test the validity of the HZ concept, future observations should measure the 2D *S*-pCO_2_ distribution of habitable, Earth-like exoplanets. This measured distribution can be compared to the distribution of habitable planets we predict in Fig. 1 to determine if Earth-like planets in the HZ are consistent with the *S*-pCO_2_ predictions of the carbonate–silicate weathering cycle.

**Fig. 3:**
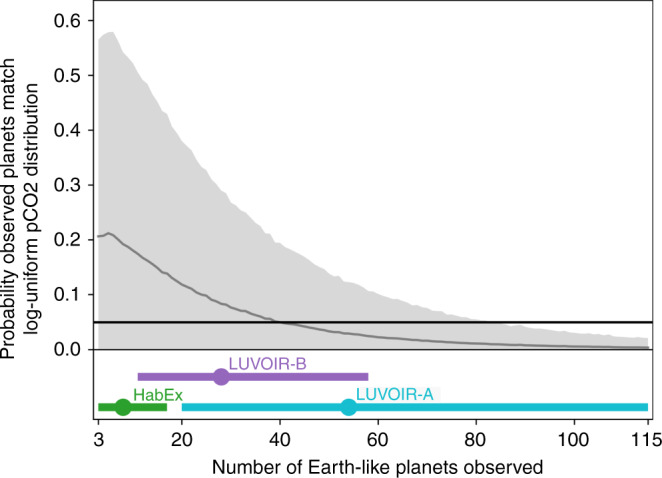
The probability observed exoplanets will accidentally match a log-uniform distribution for pCO_2_ in the HZ if the true pCO_2_ distribution is regulated by the carbonate–silicate weathering cycle, as shown in Fig. 1. This probability is shown on the vertical axis. The horizontal axis shows the number of observed Earth-like exoplanets. The solid gray curve and corresponding shaded gray region show the mean probability and 2*σ* uncertainty, respectively, that the observed planets, sampled from the planets shown in Fig. 1, match a log-uniform pCO_2_ distribution in the HZ. This curve is calculated from 10,000 two-dimensional Kolmogorov–Smirnov tests (see Results, subsection “Observational uncertainty for pCO_2_ in the HZ”). The solid, horizontal black line highlights the 5% probability line. At the bottom of the figure, the labeled points and error bars show the number of Earth-like exoplanets the next generation of proposed space telescopes are expected to observe (telescope data in Table 2). The vertical scaling of the telescope points is arbitrary, only the horizontal position and extent of the 1*σ* error bars is significant. To rule out a log-uniform pCO_2_ distribution with 95% confidence, future telescopes would need to observe at least 83 Earth-like planets.

A test of the 2D phase space of *S* and pCO_2_ in the HZ is shown in Fig. 3, which was produced using a 2D Kolmogorov–Smirnov (KS) test. The 2D KS test compares the statistical similarity of a sample distribution to a reference distribution^47–49^. For Fig. 3, the reference distribution was comprised of 500 randomly generated planets from the log-uniform distribution for pCO_2_ described above (10^−4^ ≤ pCO_2_ ≤ 10 bar, 0.35 ≤ *S* ≤ 1.05*S*/*S*_⊕_, and surface temperature between 248 and 355 K). The sample distribution was generated by randomly selecting a number of planets from Fig. 1 equal to the number of observed exoplanets. For a given number of observed exoplanets in Fig. 3, the horizontal axis, we ran the KS test 10,000 times then calculated the mean and standard deviation from those runs, shown by the gray contour and shaded region. This resampling is necessary as the 2D KS test is a nonparametric approximation that two data sets come from the same underlying population^48^. We note that below  ~20 observed planets and for probabilities above   ~0.1, the 2D KS test used here can be unreliable^49^. These limitations do not invalidate the analysis shown in Fig. 3, as we want to know, with 95% confidence, that a log-uniform pCO_2_ distribution can be ruled out if real exoplanets follow the distribution shown in Fig. 1, which corresponds to the gray line and shaded contour dipping below the 0.05 probability value, shown by the horizontal black line, at 83 observations in Fig. 3.

Thus, confidently detecting the carbonate–silicate weathering cycle will require many exoplanet observations, as shown in Fig. 3. Proposed NASA telescopes, HabEx and LUVOIR, are expected to observe between 3 and 115 Earth-like exoplanets^30,31^ (see Table 2). The ranges for each mission concept are shown by the colored circles with error bars in Fig. 3. Only the nominal capability of LUVOIR-A, the variant of the proposed LUVOIR space telescope with a primary mirror diameter of 15 m, would provide sufficient Earth-like exoplanet detections to confidently discriminate between a log-uniform pCO_2_ distribution in the HZ and a pCO_2_ distribution regulated by the carbonate–silicate weathering cycle. A caveat is that this calculation does not consider the instrument uncertainty in derived pCO_2_ measurements for each telescope or that other processes not considered in our model may alter pCO_2_ in the HZ, as discussed below.

**Table 2:** The number of expected Earth-like exoplanets observed by each platform from the HabEx^30^ and LUVOIR^31^ final reports.

Telescope	Diameter (m)	Expected yield (1*σ*)
HabEx	4	${8}_{-5}^{+9}$
LUVOIR-B	8	$2{8}_{-17}^{+30}$
LUVOIR-A	15	$5{4}_{-34}^{+61}$

## Discussion

Our model assumes that the full variation and uncertainty in Earth’s carbon cycle parameters through time (Table 1) are representative of habitable Earth-like exoplanets generally. This assumption is a reasonable first-order approximation as the bulk composition and geochemistry of rocky exoplanets appear similar to Earth’s^50^. However, the validity of this assumption likely depends on the parameter in question. For example, it is probably reasonable to expect habitable exoplanets to have a wide range of land fractions and outgassing fluxes, but it is unclear whether there is as much natural variability in the temperature dependence of silicate weathering. An improved mechanistic understanding of weathering on Earth^51,52^ might reduce these uncertainties.

Other weathering feedbacks have been proposed to operate on the Earth through time, such as reverse weathering^53^. In reverse weathering, cations and dissolved silica released from silicate weathering are sequestered into clay minerals rather than carbonates so that CO_2_ remains in the atmosphere, warming the climate and reducing ocean pH. Reverse weathering is thought to be strongly pH dependent and as ocean pH decreases, reverse weathering turns off, acting as a climate stabilization mechanism similar to the carbonate–silicate cycle. The importance of reverse weathering is so poorly constrained through Earth’s history^45^ that it does not make sense to consider it in our model. However, with future constraints from geology and lab measurements, reverse weathering might alter the stable CO_2_ abundances of our modeled atmospheres shown in Fig. 1.

At both the inner and outer edges of the HZ, our model assumes that abundant liquid water exists at the planetary surface because, without a liquid surface ocean, the carbonate–silicate weathering cycle ceases and CO_2_ cannot be sequestered after outgassing. Beyond these temperature bounds, other processes must regulate pCO_2_. This is a caveat to consider in future observations. As we see from Fig. 1, Mars has low atmospheric CO_2_ and low incident flux. Frozen exoplanets similar to Mars, populating the white area under the 248 K contour in Fig. 1, could exist in exoplanet surveys. Similarly, planets devoid of surface water, such as Venus, might exist at high pCO_2_ within the HZ. If future observations detect such planets without confirming the existence of a liquid surface or surface temperature, it could introduce additional uncertainties in any relation between orbital distance and atmospheric CO_2_. Detecting a surface ocean, one of the most important surface features to confirm when searching for biosignatures and habitability^54–56^, is also important to interpret trends of CO_2_ in the HZ.

Because we only consider variations on an Earth-like planet, our model predictions may underestimate the inherent variability in habitable exoplanetary conditions. Planets very different from the modern Earth, such as waterworlds without a carbonate–silicate weathering cycle^57^ or CH_4_-rich worlds^58,59^, could introduce additional uncertainty in an observed relationship between *S* and pCO_2_ in the HZ. Despite such uncertainties, future missions should measure the relationship between *S* and pCO_2_ in the HZ, or possibly a sharp transition in pCO_2_ at the inner edge of the HZ due to loss of surface water and subsequent shutoff of surface weathering^38,60^. A more complex model than presented here is necessary to predict such a jump in pCO_2_ at the inner edge of the HZ. However, if the carbonate–silicate weathering cycle is indeed ubiquitous, as is typically assumed in HZ calculations, then the relationship between incident flux and pCO_2_ may follow the *S*-pCO_2_ relationship predicted in Fig. 1. If no such relationship is observed, then the carbonate–silicate weathering cycle may have limited influence on planetary habitability and the limits of the conventional HZ could need revision. Alternatively, the HZ hypothesis could be incorrect and the long-term climate of HZ planets could be set by phenomena beyond those considered here.

A previous version of this work was published as part of a Ph.D. thesis^61^.

## Methods

### Habitable zone 1D climate model

We use the Virtual Planetary Laboratory (VPL) 1D radiative-convective climate model^5,62^ to generate surface temperatures for an Earth-like planet at various pCO_2_ and incident fluxes. We consider incident fluxes between 1.05*S*_⊕_ and 0.35*S*_⊕_, the HZ limits for a Sun-like star^6^, and atmospheric CO_2_ partial pressures between 10^−6^ and 10 bar. We assume the atmosphere is comprised of CO_2_ and H_2_O. If the CO_2_ partial pressure is below 1 bar, we set the initial atmospheric pressure to 1 bar and add N_2_ to the atmosphere such that the total surface pressure is 1 bar. We fix the stratospheric water vapor concentration to the modern Earth value and follow the Manabe–Wetherald relative humidity distribution in the troposphere with empirical constraints based on the modern Earth^63^.

We fit the surface temperature output, *T*_s_ in K, from the climate model with a fourth-order polynomial in $\mathrm{ln}\,({{\rm{pCO}}}_{2})$ and normalized stellar flux, as follows:

8 $$\begin{array}{l}{T}_{{\rm{s}}}\left(S,{{\rm{pCO}}}_{2}\right)=4.809-222.0X-68.44{X}^{2}-6.737{X}^{3}-0.206{X}^{4}\\ +1414XY+446.4{X}^{2}Y+44.41{X}^{3}Y+1.364{X}^{4}Y\\ -2964X{Y}^{2}-978.4{X}^{2}{Y}^{2}-98.86{X}^{3}{Y}^{2}-3.059{X}^{4}{Y}^{2}\\ +2655X{Y}^{3}+907.5{X}^{2}{Y}^{3}+92.87{X}^{3}{Y}^{3}+2.892{X}^{4}{Y}^{3}\\ -868.4X{Y}^{4}-304.6{X}^{2}{Y}^{4}-31.48{X}^{3}{Y}^{4}-0.985{X}^{4}{Y}^{4}\\ +1045Y-1496{Y}^{2}+1064{Y}^{3}-281.1{Y}^{4}.\end{array}$$

**Fig. 4:**
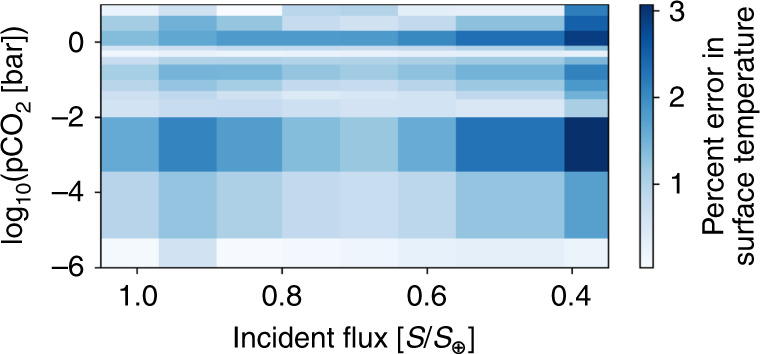
The relative error between our fourth-order polynomial fit and the full 1D radiative-convective climate model. Our polynomial fit is valid between 1.05*S*_⊕_ and 0.35*S*_⊕_, where *S*_⊕_ is the solar constant. The polynomial fit is valid for atmospheric CO_2_ abundances between 10^−6^ and 10 bar. The surface temperatures predicted by the polynomial fit reproduce the results of the 1D climate model. The maximum error in predicted surface temperature between the polynomial fit and the 1D climate model is  ~3%.

Here, CO_2_ partial pressure pCO_2_ is in bar, $X=\mathrm{ln}\,({{\rm{pCO}}}_{2})$, and *Y* = *S*/*S*_⊕_ is the incident flux, *S*, normalized to the solar constant, *S*_⊕_. Figure 4 shows the agreement between the 1D climate model and the polynomial fit used in this work.

### Numerical carbonate–silicate cycle modeling

To calculate the steady-state pCO_2_ in the atmospheres of Earth-like planets in the HZ, we use a weathering model that describes pCO_2_ on the Earth through time^21,26^. We summarize the model below and highlight how the model in this work differs from previous implementations^21,26^. These previous implementations provide a comprehensive explanation and justification of the model parameterizations, and empirical and theoretical basis. The model, as a Python script, is available in the Supplementary Data and contains a complete description of the model equations and parameters (see the file weathering_model.py).

The weathering model balances the flux of outgassed CO_2_ against the loss of carbon due to continental and seafloor weathering, which result in precipitation of carbonates in the ocean and seafloor pore space. Quantitatively, for time *t*, this is described by time-dependent equations where we normalize to the mass of the ocean, *M*_o_ (nominally, an Earth ocean, 1.35 × 10^21^ kg):

9 $$	\frac{{\mathrm{d}}C}{{\mathrm{d}}t} =\frac{{F}_{{\rm{out}}}\,+\,{F}_{{\rm{carb}}}\,-\,{P}_{{\rm{o}}}\,-\,{P}_{{\rm{p}}}}{{M}_{{\rm{o}}}}\\ 	 \frac{{\mathrm{d}}A}{{\mathrm{d}}t} =2\times \frac{{F}_{{\rm{sil}}}\,+\,{F}_{{\rm{carb}}}\,+\,{F}_{{\rm{diss}}}\,-\,{P}_{{\rm{o}}}\,-\,{P}_{{\rm{p}}}}{{M}_{{\rm{o}}}}.$$

Here, *C* is the non-organic carbon content of the atmosphere–ocean system in mol C kg^−1^, and *A* is the carbonate alkalinity in mol equivalents (mol eq). Carbonate alkalinity (henceforth alkalinity) is the charge-weighted sum of the mol liter^−1^ concentration of bicarbonate and carbonate anions, [HCO${\,}_{3}^{-}$] + 2[CO${\,}_{3}^{2-}$]. *F*_out_ is the global CO_2_ outgassing flux, *F*_carb_ and *F*_sil_ are the continental carbonate and silicate weathering fluxes, *F*_diss_ is the rate of seafloor basalt dissolution, and *P*_p_ and *P*_o_ are the pore and ocean precipitation fluxes. The fluxes on the right-hand side of Eq. (9) (*F*_out_, *F*_carb_, *P*_o_, *P*_p_, *F*_sil_, *F*_carb_, and *F*_diss_) are given in mol C yr^−1^ for d*C*/d*t* and in mol eq yr^−1^ for d*A*/d*t*.

The alkalinity that enters the ocean from weathering will balance a +2 charge cation (e.g., Ca^++^), which is why a factor of 2 enters in the definition of d*A*/d*t* in Eq. (9). Hence, geochemists often think of alkalinity in terms of the balance of cations produced in weathering, principally Ca^++^. This reasoning arises because the weighted sum of carbonate and bicarbonate concentrations must balance the charge of conservative cations minus conservative anions $\left({\mathrm{i}}.{\mathrm{e}}., \, 2[{\mathrm{Ca}}{^{++}}]\,+\,2[{\mathrm{Mg}}{^{++}}]\,+\,{\mathrm{Na}}^{+}+\ldots{\hbox{-}}[{\mathrm{Cl}}^{-}]{\hbox{-}}{\ldots}{\!}\right)$, ignoring minor contributions from weak acid anions and water dissociation. Weathering releases cations and carbon speciation adjusts to ensure charge balance, so that the cation release is effectively equivalent to carbonate alkalinity.

To improve the rate of model convergence and range of model inputs over which Eq. (9) converges, we do not consider the seafloor pore space and atmosphere–ocean as separate systems. This differs from previous versions of the model^21^, which considered the atmosphere–ocean and pore space independently. Rather, we approximate the atmosphere–ocean and pore space as a single entity in Eq. (9). This simplification does not appreciably change the model output for atmospheric CO_2_ because we run the model to steady state in all cases, where the atmosphere–ocean and pore space reach approximate equilibrium. In the next section, we present additional details on our model implementation and discuss the agreement between our no-pore model and the original, two-box model^21^.

A second modification is the range of incident stellar fluxes over which the model can be run. Previously, the model described here was used to study the Earth through time^21^ and thus only considered solar fluxes between *S*_⊕_ (the modern solar constant) and early Earth’s 0.7*S*_⊕_ (*S*_⊕_ = 1360 W m^−2^). We extend that range to include the entire conservative HZ of a Sun-like star, roughly 1.05*S*_⊕_ to 0.35*S*_⊕_^6^. We use Eq. (8), the fourth-order polynomial fit to a 1D climate model, to calculate surface temperatures throughout the HZ. The Bond albedo of the planet is calculated dynamically by the climate model and thus included implicitly in our polynomial fit.

With the coupled climate and weathering model, we generate steady-state, Earth-like planets by randomly sampling plausible initial model inputs. The ranges for each parameter we consider are representative of the Earth through time^21^ and shown in Table 1. These ranges represent very broad uncertainties of the carbonate–silicate cycle on the Earth through time and so are appropriate for Earth-like planets. We conservatively assume a uniform distribution for each parameter range shown in Table 1.

We parameterize the internal heat of an Earth-like planet conservatively using the planet’s age, ranging 0–10 Gyr, which is the approximate habitable lifetime of an Earth-like planet around a Sun-like star^64^. The equation for planetary heat relative to the modern Earth, *Q*, is given by

10 $$Q={\left(1-\frac{4.5-\tau }{4.5}\right)}^{-{n}_{{\rm{out}}}},$$

where *τ* is the age of the planet in Gyr, and *n*_out_ is the scaling exponent for internal heat, with a range given in Table 1.

The parameter ranges shown in Table 1 represent the uncertainty of the carbonate–silicate weathering cycle on the Earth through time^21^. Implicit in our assumed parameter ranges is that continental land fraction, *f*_land_, and biological weathering fraction, *f*_bio_, have increased from 0 when the Earth formed to 1 on the modern Earth. Similarly, the relative internal heat, *Q*, is assumed to be large when the Earth is young and unity for the modern Earth. Therefore, on the modern Earth, where *f*_land_ = 1, *f*_bio_ = 1, and *Q* = 1, the weathering rate is maximized and outgassing rate is relatively small (see Methods, subsection “Validity of carbon cycle parameterizations to exoplanets” for a discussion on the importance of these three parameters in our model). This is seen in Fig. 1, where the modern Earth appears near the lower bound for predicted pCO_2_ in the HZ. If the continents on an exoplanet were more easily weathered or outgassing much lower than on the modern Earth, such exoplanets could have pCO_2_ values well below the modern Earth value shown in Fig. 1. We do not consider such exoplanets in this model, so the results presented here are only applicable to planets similar to the Earth through time.

Our model assumes that each simulated planet is habitable, i.e., it has a stable, liquid surface ocean, a necessity for the carbonate–silicate cycle to operate. For a mean surface temperature below 248 K, Earth-like planets are likely completely frozen^36^, which we use as a lower temperature bound in the model. While 248 K is below the freezing point of water, it is a global mean surface temperature and 3D models show that the range 248–273 K for this parameter does not preclude the existence of a liquid ocean belt near the equator. At the other temperature extreme, a hot, Earth-like planet can rapidly lose its surface oceans due to high atmospheric water vapor concentrations that are photolyzed and subsequently lost to space. This upper temperature bound on habitability occurs at   ~355 K^37^. Above 355 K, Earth-like planets are unlikely to remain habitable for more than   ~1 Gyr^37^ and cannot operate a carbonate–silicate cycle over geologic timescales. We use these two temperature bounds, 248 K and 355 K, as the limits for habitability in our model. Any modeled planet with a final surface temperature outside these limits is uninhabitable and removed from our results.

We limit HZ planets to those with pCO_2_ below 10 bar. For most Earth-like planets in the HZ, 10 bar of CO_2_ results in planets with surface temperatures well above 355 K, which are not habitable on long time scales. If we impose a fixed stratospheric water vapor concentration in the 1D climate model and modify the tropospheric water vapor concentration based on empirical data from the modern Earth, we enable the 1D climate model to accurately model habitable, Earth-like planets through much of the HZ. But in the outer HZ, with more than  ~10 bar of CO_2_, this assumption overestimates atmospheric water vapor concentrations and leads to artificially warm planets, so we reject such cases. Above   ~10 bar of CO_2_ in the outer HZ, assuming a saturated troposphere for water vapor, increasing atmospheric CO_2_ may not lead to additional warming^6^. Rather, the surface cools in such scenarios because additional CO_2_ leads to increased Rayleigh scattering and no additional warming. Because Earth-like planets in the outer HZ would be frozen and uninhabitable even with CO_2_ partial pressures above  ~10 bar, we impose a 10-bar limit for CO_2_ in the outer HZ. This limit agrees with previous CO_2_ limitations in coupled climate and weathering models^29^.

### Combined ocean and pore space model justification

The carbon cycle model used in this work was previously derived as a two-box model^21^, where the atmosphere–ocean and the seafloor pore space were separated. In this work, we combine the ocean–atmosphere and the pore space into a single unit. This modification can be implemented in the original model^21^ by assuming that the pH of the pore space is the same as the pH of the ocean, and assuming that the alkalinity and carbon content of the ocean and pore space are the same. The dissolution and precipitation fluxes can then be calculated without treating the ocean–atmosphere and the pore space as different systems. This modification allows the model to converge quicker over a wider range of parameter combinations.

To validate our combined model, we ran the modern Earth through both the original, two-box model^21^ and our modified model at ten different incident fluxes between *S*_⊕_ and 0.7*S*_⊕_. The average error in predicted CO_2_ values between our model and the two-box model was 2.8%, with a minimum error of 2.3%, and a maximum error of 3.6%. Given the large uncertainties in model inputs (Table 1), the few percent error introduced by our simplified model is unimportant.

For each parameter combination in our simplified model, we start with the modern Earth then impose a step change for each model parameter. We then run the simulation for 10 Gyr or until the system reaches steady state. We consider the model to have reached steady state when extrapolation of the rate of change of pCO_2_ for 1 Gyr changes pCO_2_ by <1%. Typically, the model converges within a few Myr to a few tens of Myr. Rarely (2 of the 1200 planets simulated in this work), parameter combinations will not reach steady state after 10 Gyr. Simulations with combinations of exceptionally high outgassing rates and low CO_2_ weathering rates can enter a regime were atmospheric CO_2_ builds without bound, never converging. Such model results are beyond the range of validity of our model.

### Validity of carbon cycle parameterizations to exoplanets

The parameterization of weathering in our model has been empirically validated for the modern Earth^16,21,26^. The exponential temperature dependence of continental weathering is a reasonable approximation that agrees with field and lab measurements^16^ and can reproduce the climate results of more complex models^21,26^. Similarly, the power-law parameterization for the pCO_2_ dependence of continental weathering agrees with data from the modern Earth^26^ and can even be approximately derived from equilibrium chemistry arguments for an Earth-like exoplanet^38^. The bulk geochemistry of rocky exoplanets may be similar to Earth’s^50^, so we expect our weathering parameterization to reasonably approximate Earth-like planets in the HZ. However, uncertainties in how the carbonate–silicate weathering cycle regulates climate on Earth persist^21^, so the predicted variations in pCO_2_ in our model may not capture the true variability of pCO_2_ in the HZ. Below, we show that our broad parameterization of the carbonate–silicate weathering cycle may encompass the plausible range of pCO_2_ for the Earth through time, but improved understanding the carbonate–silicate weathering cycle may be necessary to know if such variations are indeed representative of the Earth through time and applicable to Earth-like planets generally.

The rate of weathering depends strongly on the intrinsic features of a planet, such as the CO_2_ outgassing rate and the properties of its continents. Changes in continental uplift rate, lithology, and configuration are parameterized in our model through the *f*_land_ and *f*_bio_ terms. The parameters *f*_land_ and *f*_bio_ linearly scale the weathering flux and could analogously be considered a continental weatherability scaling factor. For the ranges of *f*_land_ and *f*_bio_ considered in our model (see Table 1), changes in the continental weatherability alone can generate pCO_2_ values spanning  ~4 orders of magnitude. This broad parameterization likely encompasses pCO_2_ perturbations due to continental weatherability changes caused by large volcanic eruptions or changes in continental configuration. Indeed, the largest, constrained change in pCO_2_ due to such events on Earth may be closer to  ~1 order of magnitude, coeval with the eruption of the Siberian Traps^65^.

**Fig. 5:**
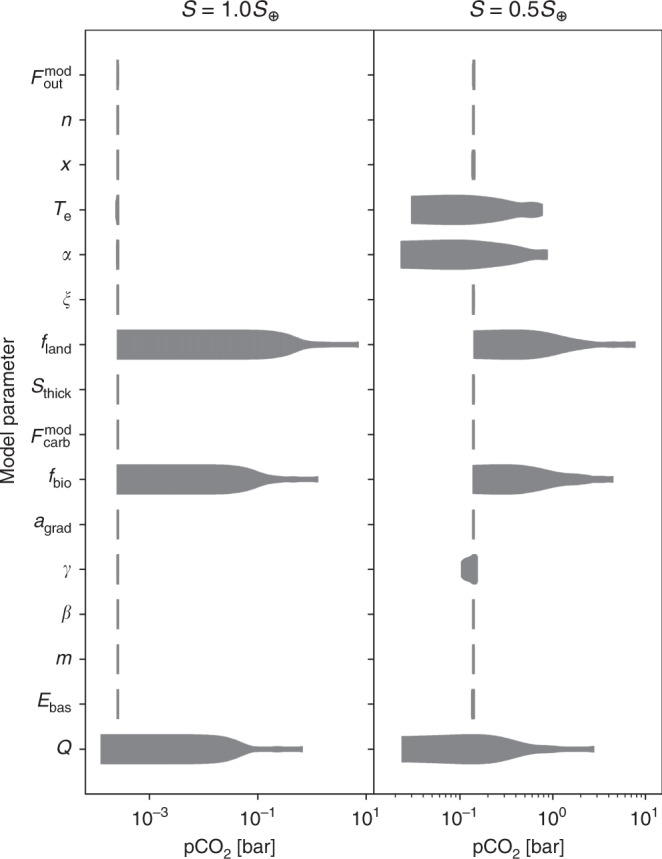
The spread in steady-state pCO_2_ from varying a single model parameter. Each parameter in Table 1 is shown on the vertical axis (note that *n*_out_ and *τ* are incorporated into *Q*, see Eq. (10)). The left panel shows an incident flux of *S* = 1.0*S*_⊕_. The right panel shows an incident flux of *S* = 0.5*S*_⊕_. For each parameter, we held all other parameters constant at the modern Earth value (see text) and randomly sampled 100 values for the parameter in question from uniform distributions of the ranges given in Table 1. The horizontal extent of the gray shaded region shows the range of possible pCO_2_ values when all other parameters are fixed. The thickness of each gray shaded region shows the relative abundance of steady-state planets at that pCO_2_. The thickest regions show maximum relative abundance, the thinnest regions show the minimum relative abundance. No surface temperature limits on habitability were imposed for the simulated planets. At low pCO_2_, three parameters (*f*_land_, *f*_bio_, and *Q*) dominate the spread in pCO_2_. At higher pCO_2_, the temperature- and pCO_2_-dependence of continental silicate weathering (*T*_e_ and *α*) are also important.

The importance of continental weatherability (*f*_land_ and *f*_bio_) on pCO_2_, relative to other parameters, is shown in Fig. 5. Figure 5 was generated by sampling uniform distributions for each model parameter shown in Table 1 across its listed range. When one parameter was varied, all other parameters were held constant at their modern Earth value, which we define as: ${F}_{{\rm{mod}}}^{{\rm{out}}}=6$ Tmol C yr^−1^, *n* = 1.75, *x* = 1, *T*_e_ = 25 K, *α* = 0.3, *ξ* = 0.3, *f*_land_ = 1, *S*_thick_ = 1, ${F}_{{\rm{carb}}}^{{\rm{mod}}}=10$ Tmol C yr^−1^, *f*_bio_ = 1, ${a}_{{\rm{grad}}}=1.075$, *γ* = 0.2, *β* = 0.1, *m* = 1.5, *E*_bas_ = 90 kJ mol^−1^, and *Q* = 1. Note that we incorporate *n*_out_ and *τ* from Table 1 into *Q*, the internal heat (see Eq. (10)), which is the parameter of interest. We show two different values for *S* in Fig. 5, *S* = 1.0*S*_⊕_ in the left panel and *S* = 0.5*S*_⊕_ in the right panel. For both values of *S*, Fig. 5 shows that variations in *f*_land_ and *f*_bio_ alone can alter pCO_2_ by orders of magnitude.

The internal heat of the planet, *Q*, plays a similarly important role in setting pCO_2_. The rate of CO_2_ outgassing is determined by *Q* and our broad parameterization of *Q* allows pCO_2_ to vary by orders of magnitude throughout the HZ, as shown in Fig. 5.

**Fig. 6:**
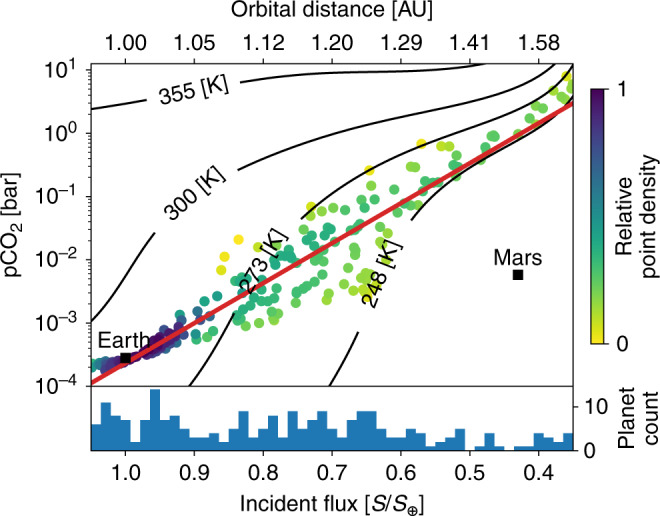
The expected distribution of stable, steady-state pCO_2_ on Earth-like planets if *Q*, *f*_land_, and *f*_bio_ are fixed to 1, i.e., modern Earth values. Except for fixing *Q* = *f*_land_ = *f*_bio_ = 1, this figure is generated identically to Fig. 1. The spread in pCO_2_ in the outer HZ is due to the temperature- and pCO_2_-dependence of continental weathering (*T*_e_ and *α*). This is expected from Eq. (4), which shows that *T*_e_ and *α* will be increasingly influential as pCO_2_ and surface temperature deviate from the modern Earth values, as discussed in the Results, subsection “Habitable zone climate theory revisited”. Without changes in *Q*, *f*_land_, and *f*_bio_, there is little spread in pCO_2_ in the inner HZ.

The rate of CO_2_ outgassing and continental weatherability drive the majority of the spread in pCO_2_ shown in Fig. 1. This is readily seen in Fig. 6, which shows the results of 300 random parameter combinations from uniform distributions of the parameters in Table 1 except for *Q*, *f*_land_, and *f*_bio_, which were all fixed to 1. Of the 300 parameter combinations, 235 remained above 248 K and are shown in Fig. 6. Comparing Fig. 6 to Fig. 1, it is readily apparent that the broad uncertainty in pCO_2_ from our results is due to variations in intrinsic planetary properties (*Q*, *f*_land_, and *f*_bio_) rather than uncertainties in the tuning parameters of our carbon cycle parameterization.

The outgassing rate and continental properties of habitable exoplanets remain unknown. Thus, our broad parameterization of those terms, which align with possible conditions on Earth throughout its history, are a reasonable approximation. If an Earth-like, carbonate–silicate weathering cycle is common on habitable planets, then these parameters may largely determine pCO_2_ on such planets and generate a range for pCO_2_ at a given orbital distance similar to that shown in Fig. 1.

## Supplementary information

Peer Review File

Supplementary Software

## Data Availability

The data used in this work are available in the Supplementary Data. Our model code depends on the location of the data directory, so the data and model are provided together in a single, zipped file.

## Code availability

The code used to generate the data and figures for this work is available in the Supplementary Data.
