# Supplementary material for: Carbonate-silicate cycle predictions of Earth-like planetary climates and testing the habitable zone concept
Source: Nat Commun. 2020 Dec 1;11:6153. doi: 10.1038/s41467-020-19896-2 (PMC7708846; doi:10.1038/s41467-020-19896-2)
Supplement: Supplementary file 2 — Supplementary Software [file 41467_2020_19896_MOESM2_ESM.zip › Code/readme.pdf]

The Python scripts and data files here can be used to reproduce the results shown in the manuscript. The DATA directory contains the random planet data we generated for this work. If you want to generate random, stable, Earth-like planets in the HZ or reproduce the main result plots of our paper, use the generate_data_and_plots.py file (look at the end of that file for examples on how to run the functions). If you want to use the coupled climate and carbonate-silicate weathering model alone, use the weathering_model.py file.

The weathering_model.py can be used without our other code and is self-contained. The comments and example at the end of that file explain how to use the model. For additional help, please email owen.r.lehmer@nasa.gov.

System requirements:
The Python code was written for use with Python 3. Other versions may work but were not tested. The required Python packages are listed at the top of each Python script. This software was run on Windows 10.

Installation guide:
Python 3 and the dependencies listed at the top of each Python file must be installed. If not already installed, these dependencies can be easily installed for free in a few minutes with a typical internet connection.

Demo:
To reproduce our results and figures, from the command line, call:
python generate_data_and_plots.py

The file is configured to generate Figure 1 when run with the above command and display it to the user. To change which figure is generated, look at the bottom of the generate_data_and_plots.py file and uncomment the desired figure. The methods to generate data are similarly commented out at the bottom of the file.

To run the coupled climate and carbonate-silicate weathering model on its own, first uncomment the lines at the bottom of the weathering_model.py file. Then, from the command line, call:
python weathering_model.py

This will run the weathering model for a single Earth-like planet at 90% Earth's incident flux. It will print the model output to the console.

Both demos should run in under 10 seconds.

Instructions for use:
The software is already configured to run on the data used in our manuscript. However, the code necessary to generate new data is included and can be run with the runMultithreadedSim() function in generate_data_and_plots.py.
